# Effects of Drought Stress on Non-Structural Carbohydrates in Different Organs of *Cunninghamia lanceolata*

**DOI:** 10.3390/plants12132477

**Published:** 2023-06-28

**Authors:** Xiaoyan Huang, Wenjuan Guo, Li Yang, Zhiguang Zou, Xinyang Zhang, Shalom Daniel Addo-Danso, Lili Zhou, Shubin Li

**Affiliations:** 1 College of Forestry, Fujian Agriculture and Forestry University, Fuzhou 350002, China; 2 Chinese Fir Engineering Technology Research Center of the State Forestry and Grassland Administration, Fuzhou 350002, China; 3 University Key Laboratory of Forest Stress Physiology, Ecology and Molecular Biology of Fujian Province, Fuzhou 350002, China; 4 Forests and Climate Change Division, CSIR-Forestry Research Institute of Ghana, Kumasi P.O. Box UP 63 KNUST, Ghana; 5 College of Geography and Oceanography, Minjiang University, Fuzhou 350108, China

**Keywords:** *Cunninghamia lanceolata* (Lamb.) Hook, plant physiology, xylem and phloem, soluble sugar, starch, non-structural carbohydrates, water stress

## Abstract

The Chinese fir *Cunninghamia lanceolata* (Lamb.) Hook. is an important timber conifer species in China. Much has been studied about Chinese fir, but the distribution of non-structural carbohydrates (NSCs) among different organs (needles, branch, stem, and roots) under drought stress remains poorly understood. In this study, we used one-year-old *C. lanceolata* plantlets to evaluate the effects of simulated drought under four water regimes, i.e., adequate water or control, light drought, moderate drought, and severe drought stress corresponding to 80%, 60%, 50%, and 40%, respectively of soil field maximum capacity on various NSCs in the needles, branch, stem and roots. The degree and duration of drought stress had significant effects on fructose, glucose, sucrose, soluble sugar, starch, and NSC content in various organs (*p* < 0.05). Fructose content increased in stem xylem, stem phloem, and leaves. Glucose and sucrose content declined in stem and branch xylem under light drought stress and moderate drought stress, and increased under severe drought stress conditions. Soluble sugars content declined, and starch content increased in leaf and branch phloem, but the latter could not compensate for soluble sugar consumption in the whole plant, and therefore, total NSCs decreased. Correlation analysis showed that a significant positive correlation existed in the soluble sugar content between leaves and roots, and between xylem and phloem in the stems and branches. Chinese fir appears to have different NSCs distribution strategies in response to drought stress, viz., allocating more soluble sugars to fine roots and increasing starch content in the needles, as well as ensuring osmosis to prevent xylem embolism. Our study may broaden the understanding of the various mechanisms that Chinese fir and other plants have to enhance their tolerance to drought stress.

## 1. Introduction

With an increase in global warming and extreme weather events, the uneven spatiotemporal distribution of precipitation will likely lead to frequent occurrences of drought stress worldwide [1]. Drought stress is one of the major abiotic stress factors that significantly influence plant survival and growth [2]. Some researchers have demonstrated that drought stress could have negative effects on morphological and physiological traits. For example, it has been shown that exposing Sargent Cherry (*Prunus sargentii*) to simulated drought lead to changes in traits via reduced leaf size and branch growth, as well as a significant decrease in maximum photosynthesis rate (*A*_max_) and stomatal conductance (*G*_s_) at *A*_max_ [3]. In addition, a severe reduction in leaf water status may inhibit the biosynthesis of chloroplast proteins and hamper chlorophyll functioning. When two cultivars of apples were exposed to drought stress, the total chlorophyll (a + b) was reduced, which eventually led to a decrease in the net photosynthetic rate (*P*_n_) [4]. Drought could destroy the photosynthetic system (reaction center or chloroplast structure), hence directly affecting photosynthetic efficiency and carbon assimilation, which reduce non-structural carbohydrates (NSCs) reserves in plants [5,6]. Often carbohydrate storage decreases when photosynthesis supply cannot compensate for growth demands, which could render plants vulnerable to carbon starvation, thereby leading to reduced forest productivity and increasing the potential for plant mortality [7]. Therefore, understanding the NSCs response mechanism of plants to drought stress is crucial for improving plant survival and growth [8].

Drought stress affects the balance in carbon supply through photosynthesis and carbon utilization for plant metabolic respiration [9,10]. Under drought stress, plants regulate the metabolic activities related to carbon assimilation through different forms of NSCs [11]. NSCs are composed of soluble sugars (such as glucose, fructose, and sucrose) and starch. The intensity and duration of drought stress can change the dynamics of the different NSCs in plant organs [12]. For example, starch in plants is consumed and assimilated into different soluble sugars, which are transported to different organs to meet the needs of physiological metabolism [13]. Furthermore, soluble sugars may play an important role as osmotic compounds to regulate water transport in plants to enhance plant drought resistance [14]. Although NSCs play a critical role in tree survival under drought stress, the association between carbon reserves depletion and drought-induced mortality in plants is still not well established [15].

Overall, most studies reported that NSCs reserves decline under short-term drought stress [16,17] but tend to increase during long-term drought simulation experiments [15,18]. Kong et al. [16] studied the effects of irrigation and fertilization on NSCs in *Eucalyptus urophylla × E. grandis* during the dry season, finding that drought stress reduced the total soluble sugar content in most of the plant organs. In contrast, ref. [18] found that both starch and sugar concentrations were generally higher after one and two years of drought stress for the slow-growing conifer *Fitzroya cupressoides*. A long-term (14 years) throughfall reduction experiment also found that total NSC concentrations generally increased in three Mediterranean tree species (*Quercus ilex* L., *Arbutus unedo* L., and *Phillyrea latifolia* L.) and that the NSC concentrations varied among different organs with the highest concentration seen in the lignotuber [15]. Drought can also affect the mobilization and transport of stored NSC reserves. For example, Sevanto et al. [13] found that the soluble sugar transport in phloem was obstructed due to severe drought stress, which influenced the distribution and utilization of carbohydrates among different plant organs, which ultimately caused carbohydrate shortage in the plants. However, the accumulation, allocation and transport of different NSC forms among different organs during drought stress need further clarification. Indeed, whether NSC pools are preferentially allocated into belowground components, including roots relative to aboveground organs, in order to enhance drought tolerance remains largely unknown.

Chinese fir [*Cunninghamia lanceolata* (Lamb.) Hook.] is a fast-growing and high-yield evergreen coniferous tree that has been planted in 19 Chinese provinces [19]. It is distributed in the entire subtropical, tropical northern margin, warm temperate southern margin, and other climatic regions in China and globally accounts for 6.5% of the total plantation area in the world [20]. In recent years, Chinese fir has suffered drought stress caused by global climate change, particularly seasonal drought in summer, which has seriously affected its survival and growth [21]. In our previous study, we stimulated polyethylene glycol (PEG)-induced drought stress for Chinese fir plantlets and found that supplying different forms of nitrogen can enhance the activities of superoxide dismutase (SOD), peroxidase (POD), and polyphenol oxidase (PPO), and nitrogen assimilation levels (e.g., glutamine synthetase (GS) and glutamate synthase (GOGAT) activity), which could alleviate the adverse effects of drought stress on Chinese fir [22]. We also found among the plant organs, fine roots (diameter < 2 mm) showed higher efficiency of water transport channels than large-diameter roots (diameter 5–10 mm), which ensures the higher water transport security of fine roots, which are critical for the uptake of water and nutrients [23]. However, to the best of our knowledge, previous studies mainly focused on the effects of drought on the growth, physiological and biochemical responses, gene regulation, and hydraulic structure characteristics in Chinese fir [23,24,25], but not on the pattern of NSC responses in different plant organs to drought.

In this context, we hypothesized that (1) drought stress would affect NSCs accumulation and distribution among different organs (needles, stem, branch, and root), (2) more NSCs would be allocated to belowground organs (roots) compared to aboveground organs to increase water uptake by the roots, and (3) drought may induce plants to preferentially transfer NSCs to fine roots, rather than to larger-diameter roots. The objectives of this study were to determine how drought stress affects the accumulation and distribution of NSCs between aboveground and belowground organs of Chinese fir, as well as the varying distribution of NSCs among different root diameter classes (fine root (diameter < 2 mm), moderate root (diameter 2–5 mm) and large root (diameter 5–10 mm)). To verify this, we used the potted water control method and determined NSC concentration in needles, branches (xylem and phloem), stems (xylem and phloem), and the roots of one-year-old Chinese fir plantlets under different drought stress intensities within 45 d. We also analyzed the correlations between NSC concentrations in the below- and aboveground organs, as well as NSC concentration between the xylem and phloem in stems and branches, to reveal the potential response mechanism of Chinese fir to drought stress. This study would provide a scientific basis for improving afforestation and forestry productivity.

## 2. Results

### 2.1. Effects of Drought Stress on NSCs in Aboveground Organs of Chinese Fir Plantlets

For aboveground organs, drought intensity reflected in the water regimes, drought duration and their interaction had significant effects on the fructose, glucose, sucrose, soluble sugar, starch, and NSC contents of Chinese fir plantlets (*p* < 0.01) (Table 1).

**Figure 1:**
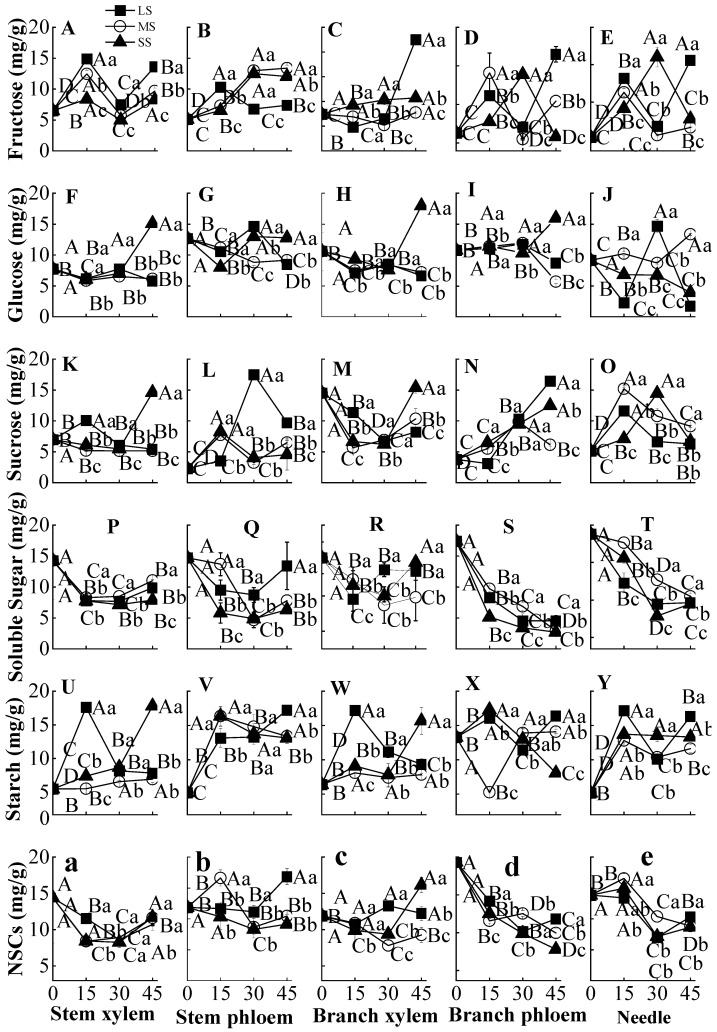
Changes in NSC content in the aboveground organs of Chinese fir plantlets under different drought stress from 0 to 45 d. (**A**–**E**) represent fructose content of stem xylem, stem phloem, branch xylem, branch phloem and needle, respectively; (**F**–**J**) represent glucose content of stem xylem, stem phloem, branch xylem, branch phloem and needle, respectively; (**K**–**O**) represent sucrose content of stem xylem, stem phloem, branch xylem, branch phloem and needle, respectively; (**P**–**T**) represent soluble sugar content of stem xylem, stem phloem, branch xylem, branch phloem and leave, respectively; (**U**–**Y**) represent starch content of stem xylem, stem phloem, branch xylem, branch phloem and needle, respectively; (**a**–**e**) represent NSC content of stem xylem, stem phloem, branch xylem, branch phloem and needle, respectively. The squares, circles and triangles represent light drought (LS), moderate drought (MS), and severe drought (SS), respectively. Different uppercase letters indicate significant differences among different durations at the same drought intensity, and different lowercase letters indicate significant differences among different intensities at the same drought duration (*p* < 0.05). Bars represent the standard deviation (*N* = 4).

At each drought duration time, the fructose content of stem xylem, stem phloem, branch xylem, branch phloem, and needles was significantly different between the three drought intensities (except for branch xylem at 15 d) (*p* < 0.05) (Figure 1A–E). On day 45, compared to control, light drought stress significantly increased the fructose content by 480.0%, 625.0%, 245.0%, and 824.2% in stem xylem, branch xylem, branch phloem, needles, respectively. The glucose content in the stem xylem, stem phloem, and branch xylem generally decreased at 15 d under different drought intensities and then increased subsequently. At 45 days, severe drought stress significantly increased glucose content by 266.8%, 254.3%, and 47.8% in the stem xylem, branch xylem, and branch phloem, respectively (Figure 1F,H,I). However, severe drought stress generally decreased glucose content in needles during different drought times and significantly decreased by 56.8% compared to the control on day 45 (Figure 1J).

For stems, severe drought stress decreased the sucrose content of stem xylem until 30 d, but then increased from day 30–45, while light drought stress significantly increased the sucrose content until 15 d but then decreased until 45 d (Figure 1K). In contrast, the sucrose content of the stem phloem under different drought intensities showed a generally increasing trend, and the sucrose content under mild drought stress was significantly higher than those under moderate and severe drought stress (Figure 1L). For the branch, the sucrose content in the branch xylem decreased for 30 d of different drought stress but then greatly increased at 45 d, whereas that in the branch phloem kept increasing throughout the experimental period (Figure 1M,N). In needles, the sucrose content showed a fluctuating trend, with an increase during early drought stress (15–30 d) and a sharp decrease at 45 d (Figure 1O).

With the extension of drought stress, the soluble sugar content and NSC contents of aboveground organs showed similar trends. Compared to the control, the soluble sugar content and NSC contents in the stem xylem, stem phloem, and branch xylem decreased for 30 days and then started to increase at 45 days (Figure 1P–R,a–c). The soluble sugar content and NSCs in branch phloem and needles showed a continuous decreasing trend compared to control, and these two organs were affected more by drought stress (*p* < 0.05) (Figure 1S,T,d,e). With the prolonged drought, the starch content of aboveground organs generally increased compared to that in control (Figure 1U–Y). The starch content of the stem phloem and needles increased more significantly during drought exposure (*p* < 0.05), and the starch content increased by 653.6%, 455.9%, and 432.80% in stem phloem under light, moderate, and severe drought stress, respectively; In needles, the starch content increased by 732.6%, 417.5%, and 536.8% in needles under light, moderate, and severe drought stress, respectively (Figure 1V,Y).

### 2.2. Effects of Drought Stress on NSCs in Belowground Organs of Chinese Fir Plantlets

With the exception of effect of plant organ on fructose (Table 1), drought intensity, drought duration and their interaction had significant effects on the fructose, glucose, sucrose, soluble sugar, starch, and NSC contents of Chinese fir plantlets (*p* < 0.01).

**Figure 2:**
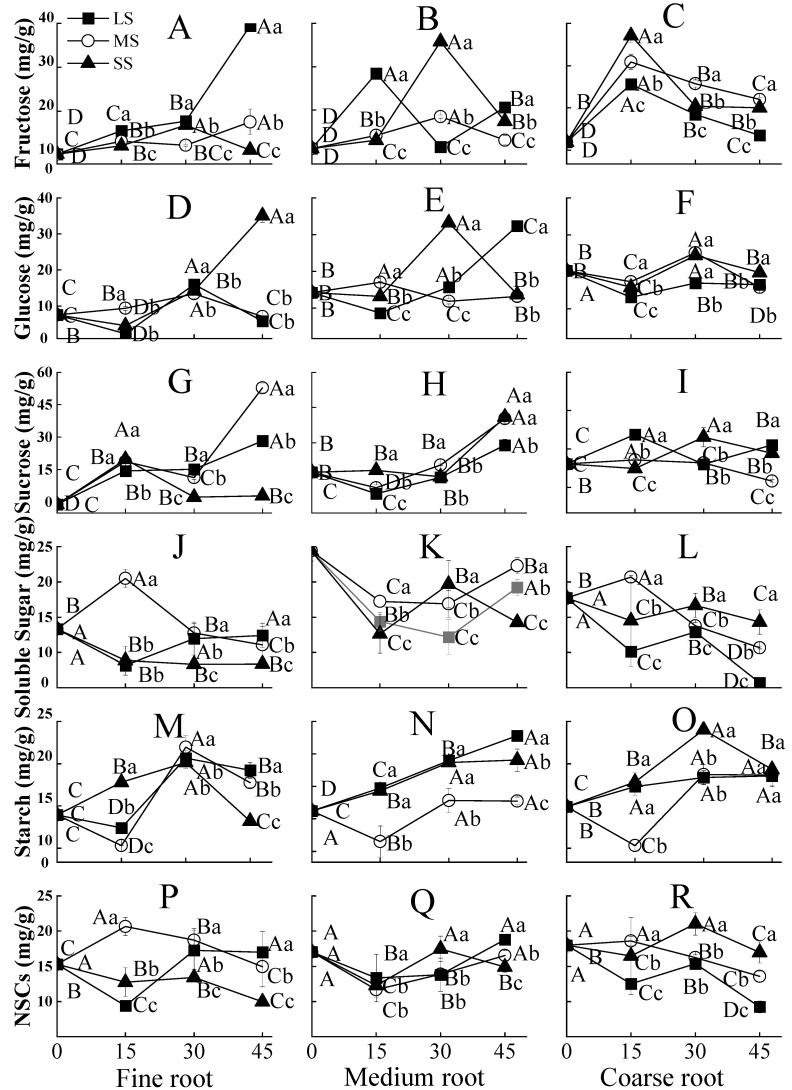
Changes in NSC content in the belowground organs of Chinese fir plantlets under different drought stress from 0 to 45 d. (**A**–**C**) represent fructose content of fine, medium, and coarse root, respectively; (**D**–**F**) represent glucose content of fine, medium, and coarse root, respectively; (**G**–**I**) represent sucrose content of fine, medium, and coarse root, respectively; (**J**–**L**) represent soluble sugar content of fine, medium, and coarse root, respectively; (**M**–**O**) represent starch content of fine, medium, and coarse root, respectively; (**P**–**R**) represent NSC content of fine, medium, and coarse root, respectively. The squares, circles, and triangles represent light drought (LS), moderate drought (MS), and severe drought (SS), respectively. Different uppercase letters indicate significant differences among different durations at the same drought intensity, and different lowercase letters indicate significant differences among different intensities at the same drought duration (*p* < 0.05). Bars represent the standard deviation (*N* = 4).

The glucose content in roots of different diameter classes generally increased during drought stress. For fine root and medium roots, it was significantly higher under light drought stress than that under moderate and severe drought stress at 45 days. For coarse roots, the glucose content was significantly lower under mild drought stress at 45 days (Figure 2A–C). Drought stress had significant effects on the glucose and sucrose content of fine and medium roots (Figure 2D–I). Specifically, at 45 days, the glucose content in fine roots was increased under severe drought compared to the response under light and moderate drought. In addition, the glucose content in the medium root was significantly higher under light drought than that under the other two drought regimes (Figure 2D,E). With an increase in drought duration, the sucrose content in fine and medium roots showed an increasing trend, but the sucrose content of coarse roots flattened (Figure 2G–I). At 45 days, moderate and light drought significantly increased the sucrose content in fine roots by 495.0% and 270.0%, compared to that in the control group, respectively, and moderate and severe drought significantly increased the sucrose content in medium roots by 135.0% and 140.0%, respectively (*p* < 0.05) (Figure 2G,H).

In the belowground organs, the soluble sugar content of the fine roots showed only a slight change, while the soluble sugar content of the medium and coarse roots significantly decreased when exposed to drought stress (*p* < 0.05) (Figure 2J–L). The soluble sugar content decreased by 5.47~29.69%, 8.54~40.73%, and 20.43~72.24% in fine, medium, and coarse roots compared to control at 45 days, respectively. Under severe drought stress, the starch content of fine roots showed an increasing trend during 30 d and then significantly decreased (*p* < 0.05). The starch content of fine roots at 45 d was slightly lower than that in control. Under light and moderate drought stress, the starch content of fine roots decreased for 15 d, then sharply increased until 30 d, and slightly decreased again until 45 days. Overall, starch content increased by 143.2% and 100.7% under light and moderate drought, respectively, compared to the control (Figure 2M). The starch content of the medium and coarse roots increased more under the light and severe drought treatments compared to the fine roots (*p* < 0.05) (Figure 2N,O). The NSC content of different root diameter classes also showed similar trends with soluble sugar; the changes in fine root NSC content were less pronounced than those in the medium and coarse roots with the extension of drought time (Figure 2P–R).

### 2.3. Correlations between Soluble Sugar, Starch, and NSC Content in Different Organs of Chinese Fir Plantlets

The fructose content of fine root and medium root was positively correlated with fructose content in the aboveground organs (Table 2). Specifically, there was a very strong positive correlation between the fructose content of fine root and the fructose content of stem xylem, branch xylem, branch phloem, and needle. Furthermore, the fructose content in the medium root was significantly and positively related to that of the stem phloem, branch phloem, and needle (*p* < 0.01). The glucose content of the fine root was strongly and positively correlated with that in the xylem and phloem in the stem and branch. The sucrose content of the fine root was negatively related to that of the stem xylem and branch xylem but correlated positively with that of the stem phloem. There was a very strong positive correlation between sucrose content in coarse roots and that in the branch and needles.

The correlation between soluble sugar, starch, and NSC contents in the different organs of Chinese fir plantlets differed significantly (Table 3). The soluble sugar content of fine roots, medium roots, and coarse roots was significantly and positively correlated with that of needles (*p* < 0.01). The soluble sugar content of medium roots was significantly positively correlated with that of stem xylem and stem phloem and negatively correlated with that of branch phloem (*p* < 0.05). The soluble sugar content of coarse roots was significantly and positively correlated with that of branch phloem (*p* < 0.01). The starch content of fine roots was weakly correlated with that of the stem and branch phloem (*p* < 0.05). The starch content of medium roots was positively correlated with that of all the aboveground organs (*p* < 0.05). In the coarse roots, starch content was weakly correlated with that of the stem xylem, branch phloem, and needles (*p* < 0.05). The NSC content of medium roots was very strongly and positively correlated with that of stem xylem but correlated negatively with that of needles (*p* < 0.05).

We also analyzed the correlations of different NSCs forms between the xylem and phloem in the stem and branch (Table 4). There was a strong correlation between fructose content in the branch xylem and branch phloem. However, there was no correlation between the fructose content of the stem xylem and the stem phloem. The soluble sugar content of the stem xylem was significantly and positively correlated with that of the stem phloem, and a similar trend was observed for the branch xylem and phloem (*p* < 0.05). The soluble sugar content of the stem xylem was significantly and negatively correlated with the starch content of the stem phloem (*p* < 0.05). The NSC content of the stem xylem was significantly and positively correlated with the NSCs and soluble sugar contents of the branch phloem and negatively correlated with the starch content of the stem phloem (*p* < 0.01).

### 2.4. Principal Component Analysis (PCA) of NSCs in Different Organs of Chinese Fir

**Figure 3:**
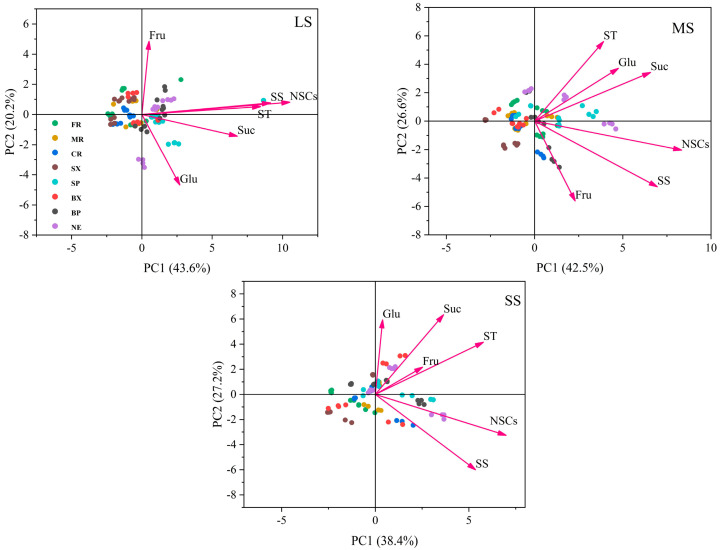
Principal component analysis (PCA) of different drought stress treatments on all indexes in different organs of Chinese fir plantlets. PC: principal component, Glu: glucose, Fru: fructose, Suc: sucrose, SS: soluble sugar, ST: starch, NSCs: non-structural carbohydrates. LS: light drought, MS: moderate drought, SS: severe drought. FR: fine roots, MR: medium roots, CR: coarse roots, SX: stem xylem, SP: stem phloem, BX: branch xylem, BP: branch phloem, NE: needles.

We used principal component analysis to test drought stress on all indexes in different organs of Chinese fir plantlets for different drought intensities (Figure 3). For light drought stress, the first two principal components (PCs) accounted for 63.8% of the total parameters, the contribution rate of PC1 was 43.6%, and the contribution rate of PC2 was 20.2%. Under moderate drought stress, the first two PCs accounted for 69.1% of all parameters, with a contribution rate of 42.5% for PC1 and a contribution rate of 26.6% for PC2. Under severe drought stress, the first two PCs accounted for 65.6% of the total parameters; the contribution rate of PC1 was 38.4%, and the contribution rate of PC2 was 27.2%. In PC1, the positive load values of soluble sugar and NSC contents were highest under the three drought stresses. Light drought had a great influence on soluble sugar, and NSC contents in stem phloem, and moderate drought had a great influence on NSC content in needles. Severe drought had a great influence on soluble sugar content in coarse roots and branch xylem, as well as on NSC content in stem phloem, branch phloem, and leaves. In PC2, the positive load value of fructose content in light drought was the largest, which affected the fructose content of each organ. The positive load value of sucrose and starch content in moderate and severe drought was the largest. A moderate drought had a greater impact on sucrose and starch content in stem phloem and leaves, and a severe drought had a greater impact on sucrose content in branch xylem, branch phloem, and leaves. The different forms of NSCs can not be separated by organs in the three drought intensities, indicating that drought had a great influence on the organs of Chinese fir plantlets.

## 3. Discussion

### 3.1. Effects of Drought Stress on NSCs Distribution in Aboveground Organs

Our results confirmed that drought stress has an effect on the accumulation and distribution of NSCs among different plant organs. For aboveground organs, the soluble sugar and NSC content generally decreased as drought stress prolonged, and these values in branch phloem and needles decreased more than those in other aboveground organs under drought stress (Figure 1S,T,d,e). This observation is consistent with the results of Zhang et al. [6], who found that the NSCs concentration of *Robinia pseudoacacia* in different organs decreased under simulated drought stress. Leaves are the main photosynthetic organs and the primary source of NSCs [26]. It is well established that drought stress could destroy chloroplast structure and can decrease photosynthesis, which inhibits NSCs synthesis in the needles. Conversely, the starch concentration increased with drought stress, and the increases were 732.6%, 417.6%, and 536.8% in light, moderate, and severe drought stress, respectively (Figure 1Y). Starch is a relatively long-term storage form of NSCs compared to soluble sugar, and it can degrade and provide the production of heat, soluble sugars, and metabolites. Starch synthesis in leaves can facilitate carbon storage and utilization, which can prevent plant injuries due to excessive respiratory consumption in response to drought [27]. Therefore, the significant increase in starch content in leaves can provide a temporal carbon sink for the conversion to soluble sugars (e.g., fructose, glucose, sucrose, etc.), thereby enhancing the resilience of plants to drought stress.

Xylem is the main water-transporting organ, and the phloem is an important channel for NSCs transport. In the present study, the fructose concentration in stem phloem and the sucrose concentrations in branch phloem showed a continuing increasing trend in response to drought stress (Figure 1B,N). This result indicates the accumulation of fructose and sucrose varied between the xylem and phloem, which may help the defense system in alleviating the damage caused by drought. Often plants produce excess reactive oxygen species (ROS) under stress, which leads to rapid injury to living tissue [28]. To compensate for the injury, plants may produce antioxidants, flavonoids, and secondary metabolites, which play an important role in detoxifying ROS and protecting the plant against any stress condition by stabilizing proteins and amino acids [28,29]. Tauzin and Giardina [30] also found that plants could produce sucrose during photosynthesis, which may be transported from source to sink tissues via the phloem—altering carbohydrate partitioning and triggering the establishment of the plant defense system.

Some studies have found that soluble sugar also plays an important role in hydraulic conductivity regulation in the xylem [31,32]. The increase in fructose in the stem phloem and the increase in the branch phloem could lead to increased phloem activity and carbohydrate transport ability during drought stress, which is beneficial for carbon allocation to other organs [33,34]. Stem xylem biomass is greater than that of other organs, and even small changes in soluble sugar content may represent large changes in the NSCs sink [35]. The increase in fructose and sucrose concentration in the stem phloem and branch phloem is also beneficial for the transport of carbohydrates to the xylem, helping to regulate the xylem osmotic potential to halt the potential for xylem embolism [36].

### 3.2. Effects of Drought Stress on NSCs Distribution in Belowground Roots

We found that the soluble sugar and NSC content of different root diameter classes generally decreased due to drought stress (Figure 2J–L,P–R), which is consistent with the results of previous studies [6,37]. However, interestingly, we found that the soluble sugar and NSC contents of fine roots showed less reduction compared to those in larger-diameter roots (medium and coarse roots) when exposed to drought. This indicates that when Chinese fir is exposed to drought, the response could be more allocation of soluble sugars and total NSCs to fine roots. These results support our third hypothesis that the accumulation of NSCs under drought stress varies among roots with different diameter classes and that there could be a shift in NSCs to smaller diameter roots.

In a previous study, we found that the water-transport efficiency of fine roots is higher than that of medium or coarse roots [23]. Water uptake varied significantly among different root diameter classes. Fine roots are the main organs for water and nutrient uptake owing to their low lignification and high specific root length, whereas coarse roots mainly play a role in fixation and support in the soil [38]. Therefore, increased distribution of soluble sugar and NSCs to fine roots could be beneficial for maintaining a higher ability for water uptake.

### 3.3. How Does NSCs Distribution in Different Organs Helps Alleviate Drought Stress?

NSCs are an important carbon pool in plants, and their production, accumulation and loss play important roles in plant drought resistance, which greatly affects plant survival and growth during drought [39]. NSCs in plants are mainly produced and stored in needles. They are then transported to stems, branches, and roots through the phloem [32,40]. PCA indicated that the sucrose, soluble sugar, starch, and NSC contents in Chinese fir were the parameters that were most influenced by drought stress, and they were mainly affected in the phloem and needles (Figure 3). Soluble sugars play an immediate role as short-term reserves in plants under drought stress, as they can be consumed directly. We found that soluble sugar contents were reduced in all the above- and belowground organs of Chinese fir plantlets in response to drought stress, but this response varied among different plant organs (Figure 1P–T and Figure 2J–L). The soluble sugars in branch phloem and needles decreased significantly compared to other aboveground organs; the soluble sugars in fine roots decreased less than those in medium and coarse roots when exposed to drought. This indicated that soluble sugar was produced by needles and was preferentially transported by phloem to fine roots, which supports our second hypothesis. Correlation analysis indicated that the soluble sugar content of needles was significantly and positively correlated with that of fine roots, medium roots, and coarse roots, respectively (Table 3), which also indicates that soluble sugar is more likely to be shifted to belowground organs in order to meet the requirement of water and nutrient uptake [38,41]. This again aligns with our second hypothesis that soluble sugar would preferentially be allocated to belowground rather than aboveground organs. Previous studies reported that roots might store soluble sugars and NSCs, which ensures that plants can reuse them to alleviate carbon starvation, and this serves as a defense against drought stress [16,42].

Starch is usually a long-term reserve, and we found the starch content increased in all above- and belowground organs, with a higher increase in stem phloem and needles (Figure 2V,Y). However, the increase in starch content may not compensate for the consumption of soluble sugar and result in a decrease in NSC content in each organ. The sucrose-unloaded hypothesis proposes that sucrose could be used as an osmotic and energy source for refilling, which would be unloaded in the direction of embolized vessels via ray parenchyma, thereby establishing a gradient to drive water movement from the phloem [43,44]. In the present study, severe drought stress significantly increased sucrose concentration in the stem xylem at 45 d (Figure 1K). In addition, different drought stress regimes increased sucrose concentration in branch phloem during the entire duration of the experiment (Figure 1N). We also found that the soluble sugar content of the xylem was significantly positively correlated with that of phloem in both stem and branch (Table 4), which further confirmed that the increase in soluble sugar content in the phloem was beneficial for the increase of soluble sugar content in the xylem. The increase in soluble sugar content in the phloem can enhance carbohydrate transport efficiency and benefit proper carbohydrate distribution in the xylem and roots [19,45]. However, which form of soluble sugar plays a dominant role in osmosis for refilling water from the phloem to the xylem in Chinese fir under drought would require further investigation.

## 4. Materials and Methods

### 4.1. Plant Material and Samplings

We used plantlets cultivated in the greenhouse of the forest-intensive breeding facility at Fujian Agriculture and Forestry University. During the cultivation period, plantlets were watered normally, maintaining the soil water content similar to the field water-holding capacity. Healthy one-year-old plantlets were selected. The plantlets were, on average, 40 cm in height and 50 mm in basal diameter. The drought stress experiment began in November 2021. Four water regimes were used to represent various drought treatments: adequate water (control, CK), light drought (LS), moderate drought (MS), and severe drought stress (SS), with 80%, 60%, 50%, and 40% of the field water-holding capacity [45]. After applying drought stress, the soil moisture content of each potted plantlet was monitored daily in a greenhouse. A portable soil moisture monitor (TZS-2X-G, Zhejiang Top Cloud-Agri Technology Co., Ltd., Hangzhou, China) was used to measure the soil moisture content of each plantlet pot. Measurement sites were the middle part of the trunk of the plantlets and the edge of the flowerpot, with a measurement depth of 8 cm [46].

After applying the drought treatments, samples were taken on days 15, 30, and 45, and destructive sampling was performed using the whole-plant harvesting method. During each sampling, four replicates of the drought treatments were collected for further measurements. The roots were divided into three diameter classes, fine roots (diameter ≤ 2 mm), medium roots (diameter 2~5 mm), and coarse roots (5–10 mm). Stem xylem, stem phloem, branch xylem, branch phloem, and needles were also collected using standardized techniques. The samples were placed in an oven at 105 °C for 15 min and dried at 80 °C until a constant weight was achieved [28]. Afterward, the samples were crushed using a grinder to determine the changes in the content of non-structural carbohydrates.

### 4.2. Measurement of Non-Structural Carbohydrate Content

After crushing the dry samples in a grinder, 0.2 g each of fine roots, medium roots, coarse roots, stem xylem, stem phloem, branch xylem, branch phloem, and needles of the Chinese fir plantlets were collected to determine the soluble sugar, glucose, fructose, sucrose, and starch contents. Approximately 0.2 g sample was placed in a 10-mL centrifuge tube, and 5 mL of an 80% (volume percentage) ethanol solution was added. After mixing, the centrifuge tube was placed in a water bath at 80 °C for 30 min, cooled to room temperature (approximately 25 °C), and centrifuged at 3500 rpm for 10 min. The supernatant was collected in a 25-mL volumetric flask. The process was repeated twice. After the volume was determined using ultrapure water, the absorbance was measured at 620 nm using the anthrone-sulfuric acid method. After that, the concentration of soluble sugars was calculated. The precipitate that was left after the extraction of soluble sugars was measured at 620 nm using the Perchloric acid method, and starch concentration was also calculated. The total NSC content was the sum of soluble sugar and starch contents. Glucose content was measured at 460 nm using an enzyme-labeling method. Sucrose and fructose contents were measured at 485 nm using the resorcinol method [36,47,48].

### 4.3. Statistical Analysis

The differences in non-structural carbohydrate indices of each organ were analyzed using analysis of variance (ANOVA) and least significant difference (LSD) multiple comparisons. Three-way ANOVA was used to examine the effects of drought stress treatments, stress duration, organs, and their interactions on the contents of fructose, glucose, sucrose, soluble sugar, starch, and NSCs. All data were tested, and they satisfied the homogeneity of variance hypothesis. The relationships between fructose, glucose, sucrose, soluble sugar, starch, and NSC contents in various organs of Chinese fir plantlets during simulated drought were analyzed using Pearson correlation coefficients. The effects of different drought stress treatments on all indices in various organs of Chinese fir plantlets were tested using Principal Component Analysis (PCA). According to the component extraction standard, the characteristic value of the PC variance is greater than one, which indicates that it is representative. All statistical analyses were performed using SPSS 22.0.

## 5. Conclusions

Chinese fir regulated NSC distribution in different organs, such as needles, stems, branches, and roots, to enhance drought resistance in plants. Soluble sugar content decreased in all organs under different drought stresses, and needles and branch phloem also decreased more significantly in response to drought. Starch content increased in all organs, and the starch content in needles and stem phloem increased the most. However, the increase in starch content was not sufficient to compensate for the consumption of soluble sugars in the whole plant; consequently, the NSC content decreased. In the belowground organs, the soluble sugar and NSC contents of fine roots changed more slightly compared to those of medium and coarse roots under drought. Correlation analysis showed a significantly positive correlation between the soluble sugar contents of needles and roots (fine, moderate, and coarse roots) and between the xylem and phloem in the stems and branches. Possibly, the soluble sugars were preferentially distributed in the roots, especially the fine roots, to maximize water uptake ability. Additionally, the soluble sugar distribution increased in the xylem and phloem of stems and branches to prevent the occurrence of xylem embolism. We propose that future research on the response of Chinese fir to NSCs distribution should be discussed in combination with photosynthesis, water transport, and other critical factors.

## Figures and Tables

**Table 1:** Three-way analysis of variance (ANOVA) of the effects of drought intensity, duration, and organ type on non-structural carbohydrates (NSCs) content in Chinese fir (*Cunninghamia lanceolata*) (*F* values).

Category	Factor	Fructose	Glucose	Sucrose	Soluble Sugar	Starch	NSCs
Aboveground	T	170.361 **	52.083 **	157.013 **	105.696 **	515.943 **	48.310 **
	D	33.455 **	26.781 **	74.731 **	5.358 **	123.806 **	4.048 *
	O	39.500 **	387.895 **	700.743 **	28.792 **	375.512 **	64.856 **
	T × D	88.139 **	86.482 **	59.958 **	2.495	48.741 **	1.554 *
	T × O	21.665 **	20.559 **	268.612 **	9.451 **	76.026 **	13.638 **
	D ×O	18.016 **	49.337 **	88.814 **	2.109 *	12.880 **	2.027 *
	T × D × O	20.793 **	37.245 **	139.497 **	1.020	48.076 **	2.226 **
Belowground	T	116.350 **	915.967 **	425.071 **	18.620 **	425.071 **	2639.884 **
	D	19.229 **	952.120 **	159.381 **	13.711 **	159.381 **	9.836 **
	O	2.987	206.071 **	166.591 **	10.933 **	166.591 **	3.584 **
	T × D	36.161 **	427.521 **	57.815 **	6.273 **	57.815 **	23.236 *
	T × O	46.699 **	328.833 **	22.360 **	5.199 **	22.360 **	3.373 **
	D × O	51.705 **	113.767 **	54.996 **	5.458 **	54.996 **	6.403 **
	T × D × O	36.639 **	386.522 **	20.080 **	3.146 **	20.080 **	10.114 **

Note: T: drought duration; D: drought intensity; O: organ. * Indicates significant differences at *p* < 0.05, and ** at *p* < 0.01.

**Table 2:** Correlation analysis of fructose, glucose, and sucrose contents between above- and belowground organs of Chinese fir plantlets. Glu: glucose, Fru: fructose, Suc: sucrose. Bold in the table caption indicates significant difference. * *p* < 0.05, ** *p* < 0.01.

Organs	Index	Stem Xylem			Stem Phloem			Branch Xylem			Branch Phloem			Needles		
		Fru	Glu	Suc	Fru	Glu	Suc	Fru	Glu	Suc	Fru	Glu	Suc	Fru	Glu	Suc
Fine root	Fru	0.520 **	−0.294 *	−0.243	0.060	−0.363 *	0.440 **	0.814 **	−0.370 *	−0.351 **	0.638 **	−0.419 **	0.695 **	0.621 **	−0.054	−0.015
	Glu	−0.321 *	0.890 **	0.436 **	0.384 **	0.421 **	0.086	0.027	0.737 **	0.289	−0.282	0.688 **	0.485 **	−0.136	−0.112	−0.041
	Suc	0.487 **	−0.418 **	−0.333 **	0.355 *	−0.608 **	0.402 **	0.205	−0.447 **	−0.391 **	0.385 **	−0.676 **	0.187	0.153	0.339 *	0.162
Medium root	Fru	0.167	−0.076	0.106	0.566 **	−0.060	−0.179	0.179	−0.247	−0.386 **	0.492 **	0.006	0.252	0.795 **	−0.577 **	0.626 **
	Glu	−0.509 **	0.028	−0.242	0.213	0.437 **	−0.058	−0.069	−0.023	−0.222	0.215	0.008	0.024	0.317 *	0.106	0.472 **
	Suc	−0.036	0.498 **	0.182	0.451 **	−0.223	0.007	0.388 **	0.422 **	0.360 *	−0.074	−0.095	0.486 **	−0.162	0.051	−0.309 **
Coarse root	Fru	0.225	−0.216	−0.070	0.270	−0.441 **	0.158	−0.142	−0.202	−0.551 **	0.098	0.101	−0.055	0.201	−0.123	0.474
	Glu	0.783 **	0.230	−0.133	0.211	0.254	−0.414 **	−0.098	0.315 *	0.226	−0.267	0.247	0.141	−0.194	0.042	0.094
	Suc	0.356 *	0.074	0.264	0.279	0.067	−0.045	0.292 *	−0.025	−0.256	0.466 **	0.225	0.322 **	0.745 **	−0.626 **	0.424 **

**Table 3:** Correlation analysis of soluble sugar, starch and NSC contents between above- and belowground organs of Chinese fir plantlets. SS: soluble sugar, ST: starch, NSCs: non-structural carbohydrates. Bold in the table caption indicates significant difference. * *p* < 0.05, ** *p* < 0.01.

Organs	Index	Stem Xylem			Stem Phloem			Branch Xylem			Branch Phloem			Needles		
		SS	ST	NSCs	SS	ST	NSCs	SS	ST	NSCs	SS	ST	NSCs	SS	ST	NSCs
Fine root	SS	0.126	−0.158	0.067	0.092	−0.334 *	−0.036	0.150	−0.271	0.048	0.282	−0.500 **	0.093	0.409 **	−0.569 **	0.196
	ST	−0.428 **	−0.230	−0.527 **	−0.054	0.332 *	0.074	−0.357 *	−0.087	−0.406 **	−0.383 **	0.432 **	−0.222	0.246	0.124	−0.202
	NSCs	−0.016	−0.250	−0.114	0.072	−0.192	−0.002	−0.026	−0.252	−0.125	0.163	−0.359 *	0.026	0.373 **	−0.657 **	0.124
Medium root	SS	0.619 **	−0.294 *	0.422 **	0.357 *	−0.237	0.199	0.181	−0.007	0.175	−0.580 **	−0.601 **	0.183	0.400 **	−0.781 **	−0.114
	ST	−0.481 **	0.430 **	−0.196	−0.622 **	0.390 **	−0.362 *	−0.558 **	0.458 **	−0.221	0.054	0.303 *	0.252	−0.576 **	0.489 **	−0.252
	NSCs	0.491 **	−0.162	0.981 **	0.072	−0.059	0.032	−0.043	0.228	0.107	0.575 **	−0.493 **	0.248	0.151	−0.700 **	−0.309 *
Coarse root	SS	0.256	−0.260	0.173	0.147	−0.420 **	−0.013	0.235	−0.268	0.147	0.497 **	−0.634 **	0.284	0.574 **	−0.797 **	0.301 *
	ST	−0.340 *	0.332 *	−0.236	−0.413 **	0.281	−0.338 *	−0.336 *	0.259	−0.257	−0.529 **	0.389 **	−0.420 **	−0.560 **	0.371 **	−0.461 **
	NSCs	0.153	−0.161	0.102	−0.049	−0.310 *	−0.183	0.061	−0.078	0.035	0.329 *	−0.500 **	0.154	0.387 **	−0.796 **	0.095

**Table 4:** Correlation analysis of fructose, glucose, sucrose, soluble sugar, starch, and NSC contents between the xylem and phloem of stems and branches of Chinese fir plantlets. Glu: glucose, Fru: fructose, Suc: sucrose, SS: soluble sugar, ST: starch, NSCs: non-structural carbohydrates. Bold in the table caption indicates significant difference. * *p* < 0.05, ** *p* < 0.01.

Organ	Index	Stem Phloem						Branch Phloem					
		Fru	Glu	Suc	SS	ST	NSCs	Fru	Glu	Suc	SS	ST	NSCs
Stem xylem	Fru	0.015	−0.412 **	0.226	0.129	0.464 **	0.370 **	0.555 **	−0.191	0.085	−0.224	0.052	−0.201
	Glu	0.176	0.486 **	−0.144	−0.096	−0.125	−0.211	−0.398 **	0.696 **	0.257	−0.092	−0.467 **	−0.222
	Suc	0.128	0.243	−0.175	−0.051	−0.141	−0.124	−0.279	0.600 **	−0.032	−0.017	−0.134	−0.054
	SS	−0.390 **	0.138	−0.323 *	0.351 *	−0.593 **	0.080	−0.231	−0.192	−0.335 **	0.583 **	0.068	0.581 **
	ST	0.449 **	0.373	−0.052	−0.265	0.241	−0.164	0.008	0.479 **	0.189	−0.427 **	−0.047	−0.425 **
	NSCs	−0.258	0.174	−0.363 **	0.281	−0.548 **	0.027	−0.244	−0.036	−0.291 *	0.472 **	0.057	0.471 **
Branch xylem	Fru	−0.064	−0.280	0.209	0.075	0.303 *	0.232	0.492 **	−0.176	0.710 **	−0.290 *	0.221	−0.216
	Glu	0.040	0.444 **	−0.247	−0.008	−0.352 *	−0.185	−0.452 **	0.631 **	0.127	0.114	−0.459 **	−0.022
	Suc	−0.220	0.356 *	−0.533 **	0.257	−0.761 **	−0.107	−0.448 **	0.203	−0.218	0.542 **	−0.100	0.494 **
	SS	−0.478 **	0.371 **	−0.011	0.279	−0.428 **	0.085	−0.099	0.188	−0.019	0.366 **	−0.149	0.310 *
	ST	0.324 *	0.160	0.023	−0.139	0.240	−0.028	0.104	0.454 **	0.404 **	−0.436 **	−0.267	−0.496
	NSCs	−0.207	0.370 **	0.110	0.151	−0.190	0.066	0.006	0.353 *	0.179	0.037	−0.246	−0.034

## Data Availability

The data for this study are available from the corresponding author upon reasonable request.
